# Brain Oxygenation During Thoracoscopic Repair of Long Gap Esophageal Atresia

**DOI:** 10.1007/s00268-016-3853-y

**Published:** 2017-01-05

**Authors:** Lisanne J. Stolwijk, David C. van der Zee, Stefaan Tytgat, Desiree van der Werff, Manon J. N. L. Benders, Maud Y. A. van Herwaarden, Petra M. A. Lemmers

**Affiliations:** 1 Department of Neonatology, University Medical Center Utrecht, Utrecht, The Netherlands; 2 Brain Center Rudolf Magnus, University Medical Center Utrecht, Utrecht, The Netherlands; 3 Department of Pediatric Surgery, University Medical Center Utrecht, Utrecht, The Netherlands; 4 Department of Anesthesiology, University Medical Center Utrecht, Utrecht, The Netherlands

## Abstract

**Background:**

Elongation and repair of long gap esophageal atresia (LGEA) can be performed thoracoscopically, even directly after birth. The effect of thoracoscopic CO_2_-insufflation on cerebral oxygenation (rScO_2_) during the consecutive thoracoscopic procedures in repair of LGEA was evaluated.

**Methods:**

Prospective case series of five infants, with in total 16 repetitive thoracoscopic procedures. A CO_2_-pneumothorax was installed with a pressure of maximum 5 mmHg and flow of 1 L/min. Parameters influencing rScO_2_ were monitored. For analysis 10 time periods of 10’ during surgery and in the perioperative period were selected.

**Results:**

Median gestational age was 35+3 [range 33+4 to 39+6] weeks; postnatal age at time of first procedure 4 [2–53] days and time of insufflation 127[22–425] min. Median rScO_2_ varied between 55 and 90%. Transient outliers in cerebral oxygenation were observed in three patients. In Patient 2 oxygenation values below 55% occurred during a low MABP and Hb < 6 mmol/L. The rScO_2_ increased after erythrocytes transfusion. Patient 5 also showed a rScO_2_ of 50% with a Hb <6 mmol/L during all procedures, except for a substantial increase during a high paCO_2_ of 60 mmHg. Patient 4 had a rScO_2_ > 85% during the first procedure with a concomitant high FiO_2_ > 45%. All parameters recovered during the surgical course.

**Conclusions:**

This prospective case series of NIRS during consecutive thoracoscopic repair of LGEA showed that cerebral oxygenation remained stable. Transient outliers in rScO_2_ occurred during changes in hemodynamic or respiratory parameters and normalized after interventions of the anesthesiologist. This study underlines the importance of perioperative neuromonitoring and the close collaboration between pediatric surgeon, anesthesiologist and neonatologist.

**Electronic supplementary material:**

The online version of this article (doi:10.1007/s00268-016-3853-y) contains supplementary material, which is available to authorized users.

## Introduction

Esophageal atresia (EA) is one of the major congenital anomalies encountered in pediatric surgery, in particular EA with tracheoesophageal fistula (TEF). The repair has always been performed by thoracotomy, but is increasingly performed by thoracoscopy [1, 2]. Since no randomized controlled trial has been done, the debate on the best method to repair the esophageal atresia still continues [3].

Long gap esophageal atresia (LGEA) is a rare form of esophageal atresia. The repair of this defect is still a challenge for the pediatric surgeon [4]. Worldwide different techniques are applied, varying from delayed repair after several months, to elongation or replacement techniques [1, 5]. Some authors have stated that an LGEA is a contra-indication for a minimally invasive approach [1]. Scarce and contradictory data are available on the effects of the installation of a low-pressure carbon dioxide (CO_2_) pneumothorax (PT) in neonates and in particular on the hemodynamics and cerebral oxygenation [2, 6–8].

Neuromonitoring of the neonatal brain can provide information on neonatal cerebral oxygenation and estimate cerebral perfusion, which can be monitored non-invasively by near-infrared spectroscopy (NIRS), measuring the regional cerebral oxygen saturation (rScO_2_) [9]. The rScO_2_ is an absolute value that reflects the venous, capillary and arterial oxygen saturation and gives insight in the oxygen delivery and consumption of the brain tissue. Shortly after birth elongation and subsequent repair of the LGEA can be performed thoracoscopically without the need for a gastrostomy. We have described the first thoracoscopic repair in 2007 [10]. As these neonates undergo repetitive procedures shortly after birth, maintenance of adequate rScO_2_ is even more critical. In this study, the effect of the CO_2_-insufflation on rScO_2_ during the consecutive thoracoscopic procedures in repair of LGEA is evaluated.

## Materials and methods

This prospective case series was performed, after approval of the institutional Medical Ethical Committee of the University Medical Center Utrecht (Utrecht, The Netherlands). NIRS monitoring is used as a standard, clinical monitoring tool in the NICU of the Wilhelmina Childrens’ Hospital, University Medical Center Utrecht.

### Patients

Patients with radiologic confirmed LGEA were eligible for this single-center prospective case series and included between May 2013 and November 2014. LGEA was considered as “any esophageal atresia that has no intra-abdominal air,” as was recently defined by a working group within the International Network of esophageal atresia during the 4th International Conference in Sydney in September 2016.

At hospital admission, arterial blood gas analyses were evaluated. Routine preoperative work-up consisted of screening for associated anomalies (VACTERL), including ultrasound of the heart and aorta to exclude right descending aortic involvement. By protocol, a peripheral arterial line—for monitoring blood pressure and regular arterial blood sampling—and venous lines were placed before surgery. A Replogle^®^ suction drain was placed in the proximal esophageal pouch to prevent aspiration. Cranial ultrasound (cUS) was performed upon admission to the NICU prior to surgery and repeated postoperatively. cUS was performed by the attending neonatologist according to a standard clinical protocol using a Toshiba Aplio Machine (Toshiba Medical Systems, Zoetermeer, The Netherlands). MRI was performed on a 3.0 Tesla whole-body Achieva system (Philips Medical Systems, Best, Netherlands). Neurodevelopment is assessed by the Griffith Mental Development Scales and the Bayley Scales of Infant and Toddler Development Test, Third Edition.

### Monitoring of cerebral oxygenation

The regional cerebral oxygen saturation (rScO_2_) was measured by near-infrared spectroscopy (NIRS) to monitor changes in the cerebral oxygenation. The rScO_2_ reflects the venous (70–80%), capillary and arterial oxygen saturation and can be reliably used as a trend monitor to detect substantial changes in cerebral oxygenation [9]. It cannot be used as a robust quantitative measurement, but it can detect substantial changes in cerebral oxygenation within the same patient. The rScO_2_ is calculated using the oxygenated hemoglobin and the total hemoglobin (oxygenated and non-oxygenated hemoglobin). The NIRS monitor (INVOS 5100-P Cerebral Oximeter; Covidien, Mansfield, Massachusetts) was used with the small adult sensor (SomaSensor^®^ no. 4100-SSA Adult/Disposable). This is a transducer containing a light emitting diode and two distant sensors that was attached to the frontoparietal side of the patients’ head. The near-infrared sensor was placed at least 6 h before surgery to obtain a baseline measurement. Parameters influencing cerebral oxygenation, including end tidal CO_2_ (etCO_2_), arterial oxygen saturation, fraction of inspired oxygen (FiO_2_) and mean arterial blood pressure (MABP) were monitored. paCO_2_, pH and hemoglobin (Hb) were checked in arterial blood gases at least once every 30 min and more frequently when deemed necessary by the anesthesiologist.

A clinical treatment guideline with a multidisciplinary team of neonatologists, pediatric surgeons and pediatric anesthesiologists in our hospital is followed to keep cerebral oxygenation stable and within the target range of 55–85%, or to avoid fluctuations of >20% from baseline measurement, by adjustments of respiratory and cardiovascular support when necessary [11]. The choice for this target range was based on the 95% CI of the cerebral oxygenation in 999 neonates, from Alderliesten et al. [12], using the same sensor. It is advised to correct a low rScO_2_ by correcting the arterial saturation, the Hb and regulating the cerebral blood flow. A low cerebral blood flow can be a consequence of—for instance—a low MAPB (<gestational age in weeks), a low paCO_2_ (<30 mmHg) and a high mean airway pressure (MAP). An rScO_2_ >85% reflects an impaired oxygen utilization, an increased oxygen supply or a disturbed cerebral autoregulation. In case the arterial saturation (range 85–95%), FiO_2_, paCO_2_ (30–60 mmHg) and MAP are out of range, these should be adjusted. Hypotension was defined as a mean blood pressure below the gestational age of the patient. Treatment started with the administration of a fluid bolus, followed by vasopressor–inotropes (dopamine) when necessary.

### Anesthesia

All patients are subjected to a standardized anesthesia protocol. For the induction of anesthesia sevoflurane (6–8% inspired concentration) was used with a 40–100% FiO_2_. After induction of anesthesia rigid tracheobronchoscopy was performed by the pediatric ENT surgeon. Muscle relaxation was applied with atracurium besylate (0.5 mg/kg) and the patient was intubated. Correct placement of the tube was verified by fiberoptic endoscopy. Thoracoscopy was performed with both lungs ventilated. In all procedures sufentanil and an oxygen/air mixture in sevoflurane were used during the maintenance of anesthesia.

### Surgical procedure

The technique of the thoracoscopic repair of long gap esophageal atresia has previously been described by Van der Zee et al. (supplemental material) [13].

### Data acquisition

All vital parameters and data on rScO_2_, measured by NIRS, were continuously monitored and stored for offline analysis using locally developed software (Bedbase/Signalbase; University Medical Center Utrecht, Utrecht, The Netherlands). For analysis, 10 representative time periods of 10 min before, during and after surgery were selected. The periods in the perioperative period of the three procedures were: baseline at the NICU or PICU (baseline), during induction of anesthesia (induction), last 10 min of induction of anesthesia (anesthesia), directly after installation of a carbon dioxide pneumothorax (insufflation 1), last 10 min of CO_2_-insufflation (insufflation 2), directly after desufflation of CO_2_ (desufflation), postoperatively after 1 h (post 1 h), 6 h (post 6 h), 12 h (post 12 h) and 24 h (post 24 h). These periods were carefully selected to measure acute changes in hemodynamics or because a steady state was expected to be reached.

## Results

**Fig. 1:**
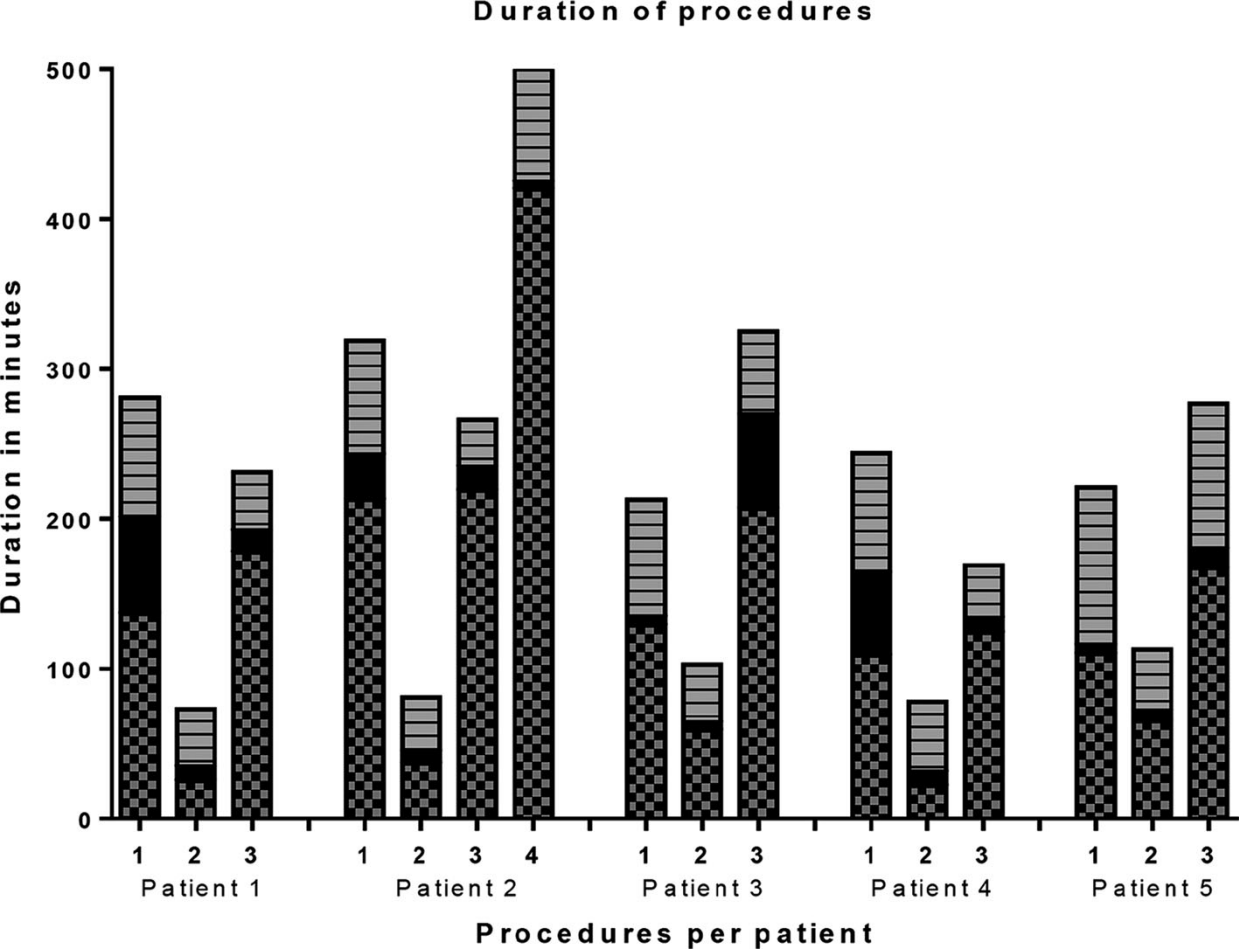
Duration of procedures. The entire duration of each procedure is displayed per patient, indicated by the *total bar*. Of each procedure the *gray part* with squares indicates the time of insufflation, the *black part* indicates the duration of surgery without insufflation of CO_2_ and the striped *gray part* the duration of induction and emergence of anesthesia, the time where no surgery is performed

Five successive patients are described in this case series with a total of 16 procedures. Patient characteristics are presented in Table 1. None of the infants had major cardiac malformations. One infant had an anorectal malformation as associated major anomaly. All infants were hemodynamically and neurologically stable before surgery. Four infants needed three consecutive surgical procedures for elongation and repair and one infant needed four procedures. The duration of CO_2_-insufflation was 127 [22–425] min (Fig. 1, Supplemental Table 1).

**Table 1:** Demographics of patients with long gap esophageal atresia

Variable	Patient				
	1	2	3	4	5
Gestational age (weeks)	34 + 0	33 + 4	35 + 2	39 + 2	39 + 6
Birth weight (g)	1580	2270	1710	2825	2570
Birth weight z-score	−1.83	0.22	−2.16	−1.76	−2.59
Apgar score (1/5/10)	9/5/8	4/7/8	5/7/8	9/9/10	4/6/9
Associated anomalies	VSD^a^ microtia	ARM^b^ tracheo malacia			
*Surgery*					
Postnatal age 1st surgery (days)	9	2	2	4	53^#^
Procedures (*n*)	3	4	3	3	3
Dilatations (*n*)	3	0	8	5	3
Mechanical ventilation (days)	15	76	14	20	13
LOS 1st admission (days)	28	78	38	43	44*
LOS 1st year (days)	42	199	74	48	156*
Lap Thal (y/n)	Y	N	Y	N	Y

*LOS* length of hospital stay

^a^Perimembranous ventricular septum defect, no treatment necessary

^b^Anorectal malformation

^#^ need for transportation from another hospital

* Length of hospital stay after transfer from another hospital, where the patient was already admitted for 52 days

### Vital parameters

**Fig. 2:**
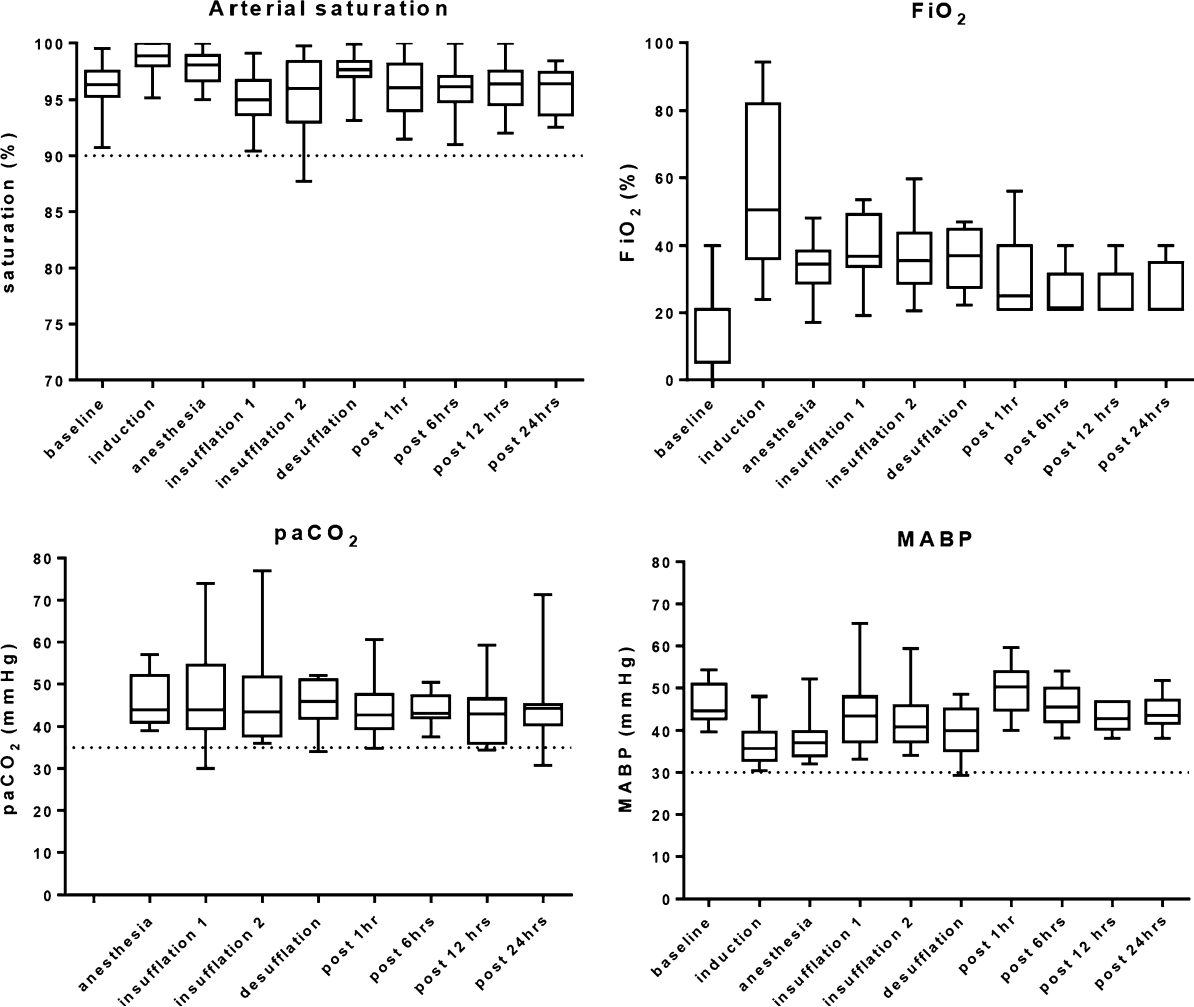
Perioperative vital parameters. Data before, during and after surgery of the vital parameters influencing the cerebral oxygenation: arterial saturation, fraction of inspired oxygen (FiO_2_), the arterial CO_2_ (paCO_2_) and the mean arterial blood pressure (MABP). The *dotted line* indicates the critical limits

**Fig. 3:**
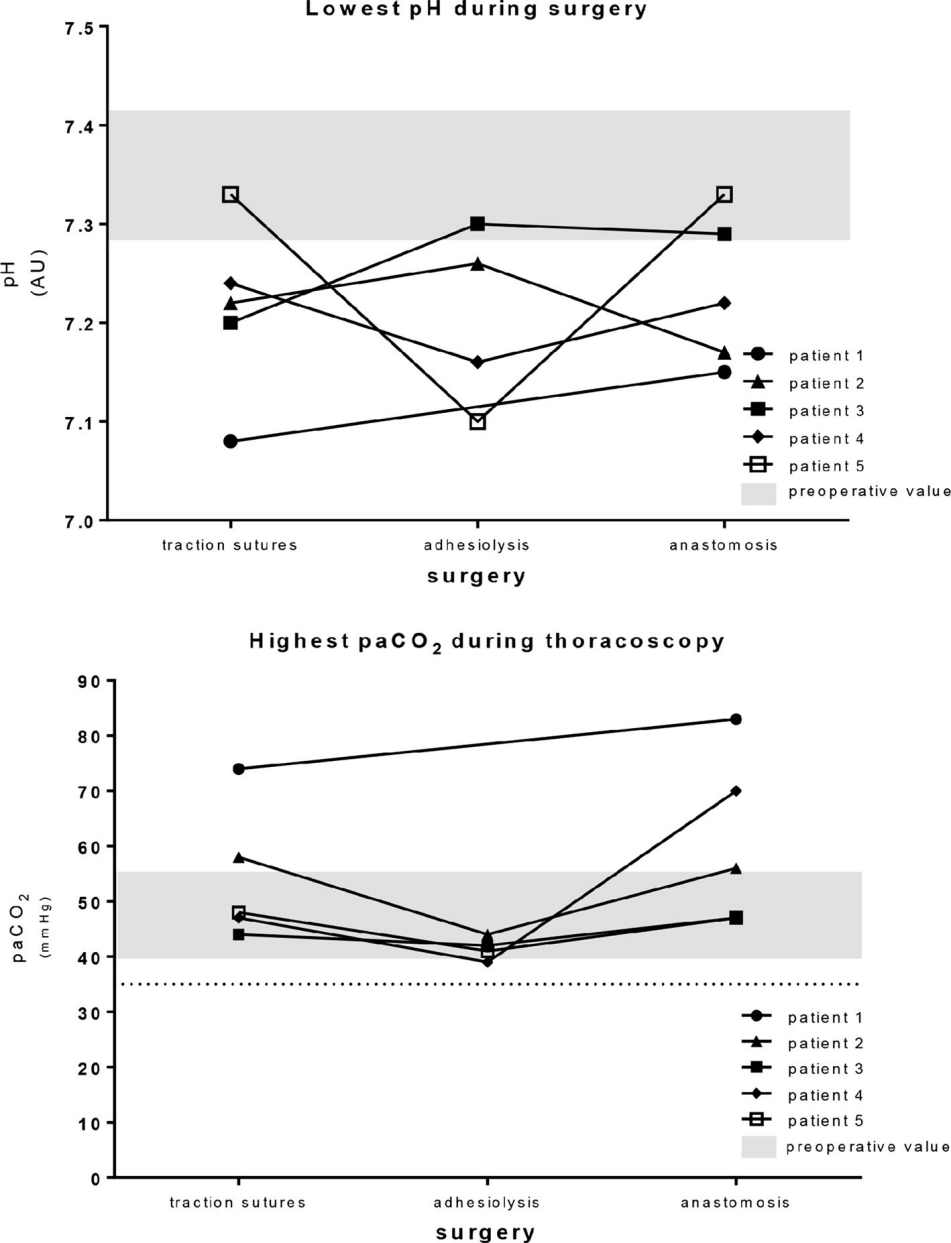
Intraoperative pH and paCO_2_. Intraoperative lowest pH and highest paCO_2_ measured during the consecutive procedures of each patient. The preoperative range of all patients is indicated by the *gray bar*. The *dotted line* indicates the paCO_2_ value of 35 mmHg

Vital parameters are shown in Fig. 2. Low arterial saturations were not observed and a high FiO_2_ was applied mainly during the induction of anesthesia. Outliers in paCO_2_ and MABP are evaluated in the following paragraph. Except for Patient 1, no severely abnormal pH and paCO_2_ values were observed (Fig. 3).

### Cerebral oxygenation

**Fig. 4:**
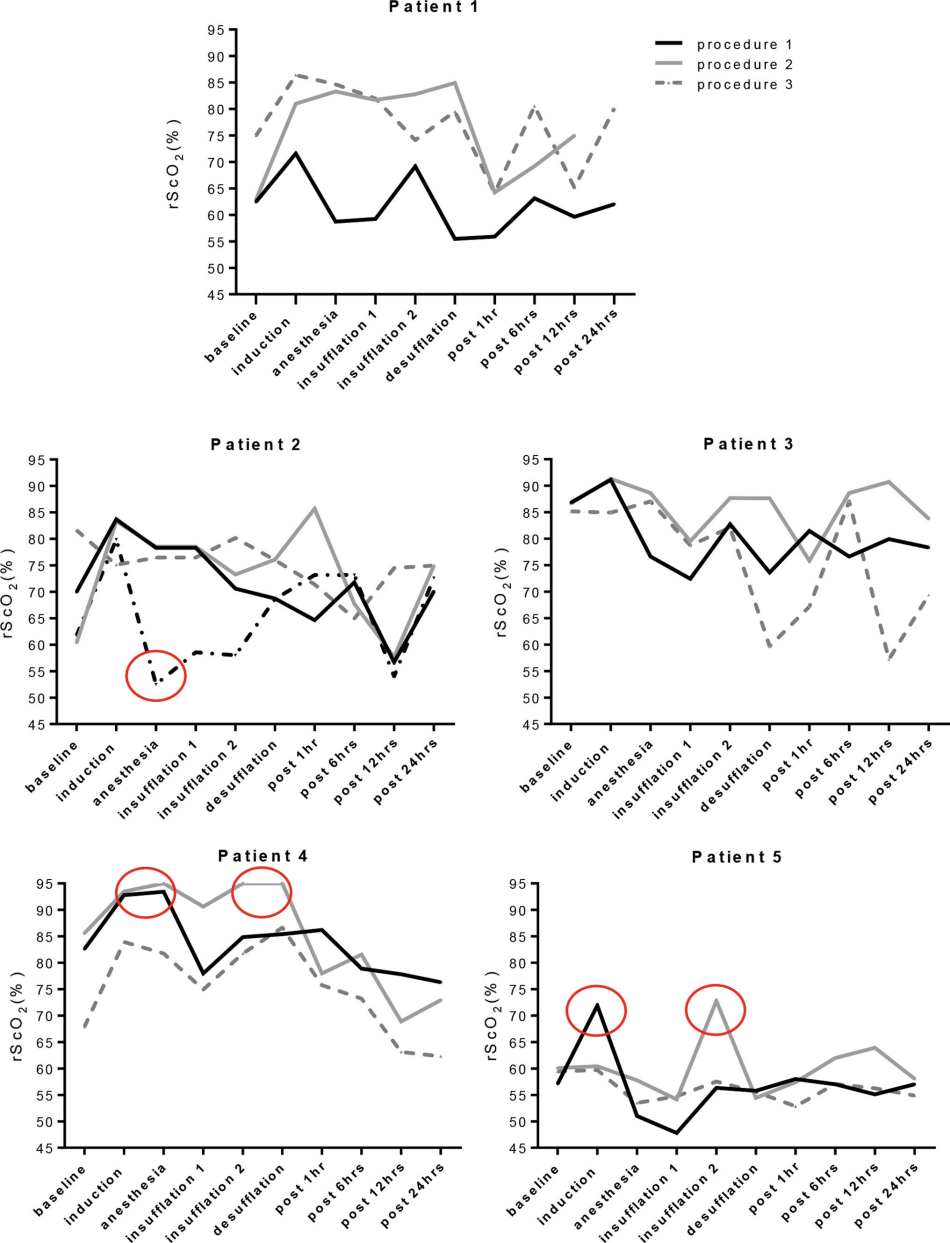
Cerebral oxygenation during consecutive long gap esophageal repair. For each patient the cerebral oxygenation in each procedure is displayed. The *black line* represents the first procedure, the *gray line* the second, the *dotted gray line* the third and *the dotted black line* the fourth procedure in Patient 2. Data are measured at 10 time periods of 10 min. The *circles* indicate the significant changes in cerebral oxygenation

Median rScO_2_ varied between 55 and 90%. In three patients (Patient 2, 4 and 5) transient outliers in the rScO_2_ (normative range (rScO_2_ 55–85%)) were observed, during all consecutive procedures (Fig. 4).

Patient 2 had a complicated, surgical course, which has been previously described in detail by Van der Zee et al. [13]. This patient was operated on four times. The third surgical procedure was performed in a semi-emergency setting due to an iatrogenic perforation of the proximal pouch after repositioning of the Replogle^®^ tube at the ward. During this re-exploration the distance between the two esophagus stumps did not approach sufficiently to perform an anastomosis, after which the traction period was continued. During the fourth procedure anastomosis could only be accomplished by a gastric pull-up. A recorded rScO_2_ value below 55% at the end of induction of anesthesia during this procedure was related to a low hemoglobin (Hb 5.1 mmol/l) and a MABP of 35 mmHg. The cerebral oxygenation increased after an erythrocytes transfusion and vasopressor support was given.

The cerebral oxygenation of Patient 4 increased above 85% during the first two procedures. This was concomitant with an FiO_2_ of 45% (35–75%, during induction of anesthesia, resulting in a paO_2_ > 100 mmHg). During the second procedure a transient mild hypercapnia of 55 mmHg occurred as well. All other parameters were within the normal range.

A low hemoglobin (Hb 5.8 mmol/l) was also encountered in Patient 5 during all procedures, with a rScO_2_ in the lower range (50–60%). His low level of hemoglobin could be explained by normal physiology (seven weeks post-term). During induction of anesthesia in the first procedure, prior to intubation, an rScO_2_ of 73% (sudden increase of 18%) was seen with a concomitant FiO_2_ of >90%. A substantial increase in rScO_2_ to 72% (increase of 19%) was seen at the end of the second procedure with an etCO_2_ of 60 mmHg.

All patients needed vasopressor support due to hypotension in at least one of the procedures (Supplemental Table 2).

Preoperative cranial ultrasonography did not show brain damage. On postoperative MRI two patients had a small thalamic infarction, and no other signs of brain damage. During follow-up (the Bayley Scales of Infant and Toddler Development, Third Edition and the Griffith Mental Development Scales) all patients showed a cognitive and motor development in the normal range.

## Discussion

This study reports on five infants with thoracoscopic repair of LGEA and the effects that these thoracoscopic procedures have on cerebral oxygenation. In this prospective case series persisting cerebral hypoxia did not occur during the consecutive thoracoscopic procedures. The infrequent transient outliers in cerebral oxygenation could be explained by a low level of hemoglobin, a decrease in MABP, an increase in CO_2_ or a high FiO_2_. Cerebral oxygenation normalized rapidly after intervention by the anesthesiologist.

The first study using NIRS in neonates that underwent thoracoscopic correction of EA and congenital diaphragmatic hernia, reported a decrease in cerebral oxygenation with severe hypercarbia and acidosis [7]. However, in this study pneumothorax pressures of up to 10 mmHg were applied. An experimental study in piglets showed that these high pressures cause severe hemodynamic instability and that these conditions could remain stable with pressures of maximum 5 mmHg [8]. As was shown in a previous study [2], cerebral oxygenation remained within the safety range (55–85%) during the single thoracoscopic correction of EA type C. The results of this current study show that by intervention of the anesthesiologist, outliers in cerebral oxygenation for the LGEA patients undergoing repetitive thoracoscopic procedures for elongation and anastomosis can be prevented or normalized quickly.

One of the factors, recognized in this study, of which the importance should be emphasized is the level of hemoglobin. A low rScO_2_ can be caused by a compromised oxygen delivery and could benefit from an increased hemoglobin [11–14], as was shown in this study. In one of the patients from this study, the low hemoglobin level was not pathological, but due to the physiological anemia of an infant. Nonetheless, this “physiological” lower hemoglobin level may compromise the cerebral saturation during major thoracoscopic surgery. High values of cerebral oxygenation were mostly due to preoxygenation. This was particularly evident in the induction phase of anesthesia, during which an increased supply of oxygen was given in order to prevent a hypoxic event while intubating the patient. This is common practice in pediatric anesthesiology. However, a high supply of oxygen can be hazardous for the neonatal brain [15].

In the present study, time periods with expected variation in vital parameters and cerebral oxygenation were analyzed. Firstly, the induction of anesthesia, with FiO_2_ between 40 and 100%, which often causes an increase in cerebral oxygenation. This increase to a maximum of 95% was observed in Patient 4. At the end of induction of anesthesia a steady state in oxygenation is expected and hypotension due to anesthetics can be observed, right before surgery is commenced. Patient 2 had a low cerebral oxygenation (52%), which could have been caused either by hypotension or low hemoglobin (Hb 5.1 mmol/l). Instillation of a CO_2_-pneumothorax can cause transient decrease in arterial saturation and hypercapnia. At the end of insufflation, either a steady state in ventilation or outliers in etCO_2_, in case of ventilation problems may be expected. In patient 5, we observed hypercapnia, with an etCO_2_ of 60 mmHg and a sudden increase in rScO_2_ to 73%, since carbon dioxide causes an increase in cerebral perfusion by vasodilatation [16, 17]. This could have been prevented by increasing the ventilation frequency settings adequately.

Directly after desufflation of the pneumothorax, a higher positive expiratory end pressure is applied to maximally expand the lung, leading to extra oxygen in the patient, which can cause an increase in rScO_2_. Desufflation in our population, however, resulted in variable changes in cerebral oxygenation. In Patient 2 an increase in both arterial saturation and MABP led to an increase in rScO_2_. Patient 1 and 3 had a small decrease in etCO_2_ with a decrease in rScO_2_. However, the exact opposite occurred in Patient 4 and 5. Significant differences in cerebral oxygenation during the different procedures within one patient were only seen in Patient 2. He had the first three procedures in the first week of life and the gastric pull-up procedure after four weeks. As this study shows, important parameters influencing the cerebral oxygenation and perfusion are arterial oxygen saturation, CO_2_, MABP, FiO_2_ and hemoglobin.

Outliers in cerebral oxygenation may pose a risk for brain injury in the developing neonatal brain [18]. From cardiac patients, undergoing major neonatal surgery, it is known that in a high percentage de novo brain injury is visible postoperatively on MRI [19]. In this study, two small thalamic infarctions were found, for which no long-term consequences are expected. Since these abnormalities were not visible at the preoperative ultrasound, they most likely originated in the perioperative period. However, these two patients did not show periods of hypoperfusion of the brain, due to hypotension, hypocarbia or a low cerebral oxygenation. In other words, the etiology of these thalamic infarcts remains unclear. To date, the prevalence of cerebral damage in patients with long gap esophageal atresia and neonatal non-cardiac surgery in general is unknown. The incidence of cerebral injury may be considerable, since a delay in neurodevelopment in patients with esophageal atresia has been reported in several studies [20, 21], which could not be explained by their primary anomaly or a genetic syndrome. In the current study, neurodevelopmental follow-up will be performed at the age of two years with the Bayley Scales of Infant and Toddler Development.

A limitation of this case series of LGEA is the small sample size of five cases. This is of course due to the fact that LGEA is a rare congenital anomaly. Patients were born at different gestational ages, both preterm and full-term, which makes their degree of vulnerability diverse. Furthermore, one of the patients underwent the thoracoscopic LGEA correction after the neonatal phase (at 58 days postnatal age) due to transfer from another hospital. For these reasons we decided to describe these patients in a case series, without applying statistics. Stressing risk factors and important time periods of fluctuations in vital parameters was the main goal of this study. This case series is the first description of cerebral oxygenation and perfusion in the thoracoscopic, repetitive procedure of LGEA, and further studies are recommended to ensure our findings. Since NIRS monitoring is part of standard clinical care in our hospital, this was a prospective study where we aimed to keep the cerebral oxygenation within the normal range. In case of outliers the anesthesiologist intervened directly and results of intervention could be ascertained instantaneous.

Thoracoscopic elongation and anastomosis of long gap esophageal atresia is feasible and makes treatment in the neonatal period possible. Consequently, the need for a gastrostomy is avoided, as is an extended hospitalization. Since these patients are at risk of developing fluctuations in vital parameters and cerebral oxygenation during surgery, they should be monitored closely. We emphasize the importance of structural neuroimaging perioperatively, to assess acute brain injury and to compare surgical methods using this outcome. Acknowledging specific time periods of risk regarding hemodynamics and cerebral oxygenation of the neonate and ensuring a close collaboration between neonatologists, anesthesiologists and pediatric surgeons is essential and makes successive thoracoscopic procedures with stable physiological conditions and cerebral oxygenation feasible.

## Electronic supplementary material

Below is the link to the electronic supplementary material.

Supplementary material 1 (DOCX 16 kb)

Supplementary material 2 (DOCX 14 kb)
