# Supplementary material for: Brain Oxygenation During Thoracoscopic Repair of Long Gap Esophageal Atresia
Source: World J Surg. 2017 Jan 5;41(5):1384–92. doi: 10.1007/s00268-016-3853-y (PMC5394154; doi:10.1007/s00268-016-3853-y)
Supplement: Supplementary file 1 — Supplementary material 1 (DOCX 16 kb) [file 268_2016_3853_MOESM1_ESM.docx]

# Supplemental table 1. Perioperative and postoperative course

| **Variable** | **Patient** | | | | |
| --- | --- | --- | --- | --- | --- |
|  | **1** | **2** | **3** | **4** | **5** |
| **Proximal fistula (y/n)** | n | y | n | n | n |
| **Gastrostomy (y/n)** | n | y | n | n | y |
| **Gastropexy (y/n)** | y | y | y | y | y |
| **Aortapexy** | n | n | n | n | y |
| **Complications** | Pneumothorax  Line sepsis | Perforation by replogle |  | Fever due to leakage  Line sepsis |  |
| **Postoperative leakage (n/y)** | n | n | n | y | n |

# Supplemental table 2. Need of blood pressure support during surgical procedure

| **Variable** | **Patient** | | | | |
| --- | --- | --- | --- | --- | --- |
|  | **1** | **2** | **3** | **4** | **5** |
| **1^st^ surgery** | dopamine | dopamine | - | dopamine | dopamine |
| **2^nd^ surgery** | dopamine | dopamine | fluid expansion | fluid expansion | - |
| **3^rd^ surgery** | dopamine | dopamine | dopamine | dopamine | dopamine |
| **4^th^ surgery** | NA | dopamine | NA | NA | NA |

*NA* Not applicable
