# Supplementary material for: Brain Oxygenation During Thoracoscopic Repair of Long Gap Esophageal Atresia
Source: World J Surg. 2017 Jan 5;41(5):1384–92. doi: 10.1007/s00268-016-3853-y (PMC5394154; doi:10.1007/s00268-016-3853-y)
Supplement: Supplementary file 2 — Supplementary material 2 (DOCX 14 kb) [file 268_2016_3853_MOESM2_ESM.docx]

## Surgical procedure

The technique of the thoracoscopic repair of long gap esophageal atresia has previously been described by Van der Zee et al.(1). All patients were operated by the same team of surgeons (DZ and ST). By standard protocol, the first procedure started with a rigid tracheobronchoscopy to exclude the presence of a proximal fistula and to determine the grade of concurrent tracheomalacia. Then the patient was positioned in a left laterally recumbent position and tilted 10-20° reverse Trendelenburg. A 5 mm intercostal camera-trocar was placed 1 centimeter anterior and below the tip of the scapula. The CO_2_-pneumothorax was installed by insufflation with a pressure of 3-5 mmHg and a flow of 1 L/min. In case of a proximal fistula, this was closed first, thoracoscopically. Mobilization of the proximal and distal esophagus was then started. Traction sutures Vicryl 4 x 0 (Ethicon, Johnson & Johnson, Amersfoort, NL) were applied, at four corners of the proximal and distal esophageal stump. The sutures were crosswise withdrawn and fixed externally with mini-mosquito forceps. Close to both ends of the esophagus, a clip was applied to the sutures to be able to assess the approximation radiographically after surgery. Then a laparoscopic gastropexy was performed trough the umbilicus against the ventral abdominal wall, to prevent the stomach from migrating into the thorax.

During the traction period, the patient remained intubated and sedated. The movement of the traction sutures was checked once daily by x-ray examination. Normally, after approximately three days no more progression could be determined. The second thoracoscopic procedure was to release the adhesions between the esophagus and lung, after which traction was installed again. When the clips had approximated sufficiently, the patient could undergo a delayed primary anastomosis. During this third surgery, the proximal and distal esophagus were opened and the two ends were advanced by the sliding suture technique with the use of slipping knots(2). The anesthesiologist placed a 6-8-Fr nasogastric tube past the anastomosis into the distal esophagus and stomach, and the anastomosis was completed. A chest-tube was only put in place if leakage was expected.

A contrast study of the esophagus was performed at day five. Oral feeding was started if no leakage was observed.

Most patients needed a laparoscopic fundoplication at a later stage. All children received an H_2_-receptor antagonist postoperatively for three months.

Reference List

1. van der Zee DC, Gallo G, Tytgat SH. Thoracoscopic traction technique in long gap esophageal atresia: entering a new era. Surg.Endosc. 2015.

2. van der Zee DC, Gallo G, Tytgat SH. Thoracoscopic traction technique in long gap esophageal atresia: entering a new era. Surg.Endosc. 2015.
